# Deregulation of Serum MicroRNA Expression Is Associated with Cigarette Smoking and Lung Cancer

**DOI:** 10.1155/2014/364316

**Published:** 2014-10-20

**Authors:** Jinkun Huang, Jianjun Wu, Yuanqi Li, Xun Li, Ti Yang, Qiaoyuan Yang, Yiguo Jiang

**Affiliations:** ^1^State Key Laboratory of Respiratory Diseases, Institute for Chemical Carcinogenesis, Guangzhou Medical University, 195 Dongfeng Road West, Guangzhou 510182, China; ^2^The First Affiliated Hospital of Guangzhou Medical University, Guangzhou 510120, China

## Abstract

Lung cancer is the leading cause of cancer-related death and cigarette smoking is the main risk factor for lung cancer. Circulating microRNAs (miRNAs) are considered potential biomarkers of various cancers, including lung cancer. However, it is unclear whether changes in circulating miRNAs are associated with smoking and smoking-related lung cancer. In this study, we determined the serum miRNA profiles of 10 nonsmokers, 10 smokers, and 10 lung-cancer patients with miRCURY LNA microRNA arrays. The differentially expressed miRNAs were then confirmed in a larger sample. We found that let-7i-3p and miR-154-5p were significantly downregulated in the sera of smokers and lung-cancer patients, so the serum levels of let-7i-3p and miR-154-5p are associated with smoking and smoking-related lung cancer. The areas under receiver operating characteristic curves for let-7i-3p and miR-154-5p were approximately 0.892 and 0.957, respectively. In conclusion, our results indicate that changes in serum miRNAs are associated with cigarette smoking and lung cancer and that let-7i-3p and miR-154-5p are potential biomarkers of smoking-related lung cancer.

## 1. Introduction

Lung cancer continues to be the most common type of cancer and the leading cause of death in economically developed countries and the second leading cause in developing countries [1]. Non-small-cell lung cancer (NSCLC) accounts for approximately 80%–85% of all cases of lung cancer [2]. Many studies have demonstrated that cigarette smoking is the primary risk factor for the development of lung cancer. Despite many efforts to reduce smoking in the population and to improve treatment measures for patients with lung cancer, the overall 5-year survival rate for NSCLC patients remains a disappointing 15% [3]. An explanation for this poor outcome is that the prognosis for patients with advanced NSCLC remains poor because the cancer is detected at an advanced and typically untreatable stage [4]. Therefore, it is crucial for the outcomes of patients with NSCLC that early and more-accurate diagnostic biomarkers are identified.

MicroRNAs (miRNAs) are a class of short, highly conserved noncoding RNAs of 18–25 nucleotides that regulate gene expression by incompletely base-pairing with a complementary sequence in the 3′-untranslated region (UTR) of the target mRNA [5]. Many studies have shown that miRNAs play important roles in numerous developmental processes, including cell proliferation, apoptosis, and tumorigenesis [6, 7]. Numerous studies have suggested that miRNAs undergo aberrant regulation during tumorigenesis [8, 9]. In lung cancer, it has been shown that the miRNA expression profiles and specific miRNAs in the lung tissue correlate with disease prognoses and clinical outcomes [10, 11]. miRNA analysis provides a more comprehensive understanding of the pathogenesis of lung cancer. The recent identification of stable miRNAs in serum has paved the way for their use as novel biomarkers in clinical diagnoses [12]. Since the discovery of serum miRNAs, much evidence has demonstrated that these miRNAs are stable and noninvasive biomarkers of a variety of cancers, such as breast cancer [13], colorectal cancer [14], and lung cancer [15]. With many advantages, such as their noninvasive analysis, high tissue specificity, stability, low complexity, and predictive utility, circulating miRNAs are considered ideal diagnostic biomarkers and antitumor drug targets [16].

Many studies have shown that there is a direct dose-response relationship between the number of cigarettes smoked and the risk of lung cancer [17, 18]. Several miRNAs have recently been shown to be dysregulated in the lungs of rats exposed to environmental cigarette smoke (ECS) and in the bronchial epithelia of smokers [19, 20]. Clarifying the dynamics and carcinogenic mechanisms behind the cigarette-smoke-induced dysregulation of miRNAs will contribute to the development of more-effective lung-cancer diagnoses, chemoprevention, and therapies [21]. Therefore, microRNA profiles might have utility as biomarkers of smoking-related lung diseases and clarify the regulatory mechanisms that mediate the host response to exposure to tobacco smoke and other environmental agents.

A recent study showed that cigarette smoking induces changes in plasma miRNA profiles in healthy subjects [22]. Another study showed that the characteristics of lung cancers in never-smokers are distinctly different from those in smokers [23]. As yet, there have been relatively few reports of the changes in serum miRNAs in cigarette-smoking-induced lung cancer. In the present study, we profiled the serum miRNAs differentially expressed in the normal, cigarette-smoking, and lung-cancer-affected populations with an miRNA array analysis to explore the relationship between serum miRNAs, cigarette smoking, and lung cancer. The expression of let-7i-3p and miR-154-5p was significantly downregulated in the sera of smokers and lung-cancer patients. A negative association was also observed between smoking and the expression of serums let-7i-3p and miR-154-5p. Dysregulated levels of miRNAs let-7i-3p and miR-154-5p are potential biomarkers of lung cancer and may play important roles in cigarette-smoke-induced lung cancer.

## 2. Materials and Methods

### 2.1. Study Design and Samples

The study was divided into three phases. In the first phase, we collected serum samples from 10 nonsmokers, 10 smokers, and 10 lung-cancer patients to identify the serum miRNAs associated with smoking and lung cancer. The expression profiles of the serum miRNAs of these three groups were compared. According to the screening criteria that are specified in the Results Section, we selected some of the differentially expressed miRNAs as candidate miRNAs for further study. In the second phase, using quantitative real-time reverse transcription-polymerase chain reaction (qRT-PCR), the levels of the candidate miRNAs were quantified and validated in an increased number of individual serum samples (30 nonsmokers, 30 smokers, and 30 lung-cancer patients). Those candidate miRNAs whose expression was consistent with the microarray profiles were selected for further analysis. In the third phase, the miRNAs were identified and confirmed in a larger set of individual serum samples (65 nonsmokers, 100 smokers, and 84 lung-cancer patients). The relationships between the levels of the candidate serum miRNAs, the smoking index, and the lung-cancer type were analyzed.

All study samples were obtained from The First Affiliated Hospital of Guangzhou Medical University, Guangzhou, China. Because most smokers in China are male, all subjects in this study were selected from the male population. The detailed smoking histories of the smokers and lung-cancer patients were collected, including their ages when they began smoking, their ages when they stopped smoking, numbers of cigarettes smoked per day, numbers of years that they smoked, and pack-years (number of cigarette packs per day × number of years smoked). The smoking index was measured in terms of pack-years smoked.

To exclude the effects of other factors in this study, we established inclusion and exclusion criteria for the study subjects. Subjects with significant second-hand cigarette exposure, respiratory diseases other than lung cancer, and exposure to occupational carcinogens were excluded from the study. According to a previous study [24], the expression of the most downregulated microRNAs in smokers was restored one week after smoking cessation. To explore the changes in serum miRNAs induced by smoking, we selected “current smokers” as subjects who had been exposed to cigarette smoke for a long time. A “regular smoker” was defined as someone who had smoked more than one pack-year, and a “current smoker” was defined as a regular smoker who still smoked in the year of the interview or in the year preceding it [25]. Subjects who had smoked but had not accumulated one pack-year were considered “occasional smokers” and were classified as nonsmokers in this study. All lung-cancer patients were newly diagnosed with a histopathological examination, and none of the patients had received any preoperative chemotherapy or radiotherapy. The study was approved by the Ethics Committee of Guangzhou Medical University. All subjects participated voluntarily in the study and gave their informed consent.

### 2.2. Collection of Human Sera and Total RNA Extraction

About 5 mL of peripheral blood was collected from each subject in a sterile polyolefin tube without anticoagulant. The blood samples were maintained at room temperature for at least 30 min and then centrifuged at 1300 ×g for 15 min at 4°C. The serum was transferred to a fresh tube and stored at –80°C until RNA extraction. Total RNA was extracted from the serum samples using the miRNeasy Mini Kit (Qiagen, Hilden, Germany), according to the manufacturer's supplementary protocol. The quality and quantity of RNA were measured with a NanoDrop spectrophotometer (NanoDrop Technologies, Wilmington, DE, USA).

### 2.3. miRNA Arrays Analysis

To investigate the differential expression of serum miRNAs among the nonsmoker controls, smokers, and lung-cancer patients, their serum miRNA expression profiles were determined using the miRCURY LNA microRNA array version 16.0 (Exiqon, Vedbaek, Denmark). This array contains more than 1891 capture probes, covering all human, mouse, and rat miRNAs annotated in miRBase 16.0. Briefly, equal volumes of serum from the 10 nonsmoker controls, 10 smokers, or 10 lung-cancer patients were pooled and the total RNA was extracted from the pooled samples of the three groups. The miRNAs were labeled using the miRCURY Hy3/Hy5 power labeling kit (Exiqon), according to the manufacturer's guidelines. After the labeling procedure was terminated, the Hy3-labeled samples were hybridized according to the instructions for the miRCURY LNA microRNA array. Following hybridization, the slides were washed several times with wash buffer (Exiqon) and dried by centrifugation for 5 min at 1200 ×g. The slides were then scanned using the Axon GenePix 4000B microarray scanner (Axon Instruments, Foster City, CA, USA). The scanned images were imported into the GenePix Pro 6.0 software (Axon Instruments) for grid alignment and data extraction. The replicated miRNAs were averaged and those miRNAs with intensities exceeding 50 in all samples were chosen to calculate the normalization factor. The expression data were normalized using median normalization. After normalization, the differentially expressed miRNAs were identified with fold-change filtering. Hierarchical clustering was performed with the MEV software (v4.6, TIGR).

### 2.4. Quantification of miRNA Expression with qRT-PCR

The expression of eight serum miRNAs (see Results Section) was quantified with qRT-PCR. All procedures were performed according to the manufacturer's instructions, with minor modifications. Briefly, 20 ng of total RNA was reverse-transcribed with the TaqMan microRNA reverse transcription kit (Applied Biosystems, Foster City, CA). The expression levels of the miRNAs were determined with qRT-PCR using the TaqMan microRNA assay kit (Applied Biosystems) with the ABI 7900 real-time PCR system (Applied Biosystems). The conditions for the reactions were those recommended by the manufacturer (95°C for 10 min, followed by 40 cycles of 95°C for 15 s and 60°C for 1 min). The cycle threshold (Ct) values were calculated with the SDS 2.0.1 software (Applied Biosystems). The average expression levels of the serum miRNAs were normalized to the average for miR-16 using the 2^−ΔΔCt^ method, as in our previous study [26]. All experiments were performed at least three times in triplicate.

### 2.5. Statistical and Bioinformatic Analyses

Statistical analyses were performed with the SPSS 16.0 software (SPSS Inc., Chicago, IL). Comparisons of the serum miRNA expression levels in the nonsmoker controls, smokers, and lung-cancer patients were made with Kruskal-Wallis one-way ANOVA. Pearson's bivariate correlation analysis was used to evaluate the correlations between the smoking index and the levels of serum miRNAs. To evaluate the predictive value of serum miRNAs, receiver operating characteristic (ROC) curves were constructed and the areas under the ROC curves (AUCs) were calculated to better identify potential serum microRNAs that could be used as biomarkers for smoking-related lung cancer. The AUC is usually considered an effective measure of the validity of a diagnostic biomarker (an AUC close to 1 indicates that the diagnostic efficiency of the biomarker is near perfect). *P* values < 0.05 were considered statistically significant.

To explore the potential functions of these candidate miRNAs during lung carcinogenesis, we predicted their target genes and the related pathways. The miRNA target genes were predicted with the miRBase (http://www.mirbase.org/), Miranda (http://www.microrna.org/microrna/), and TargetScan (http://www.targetscan.org/) online software for target gene prediction. Pathway enrichment analyses of the predicted miRNA target genes were performed with KEGG pathway (http://www.genome.jp/kegg/).

## 3. Results

### 3.1. Serum miRNA Expression Profiles of Nonsmokers, Smokers, and Lung-Cancer Patients

**Figure 1:**
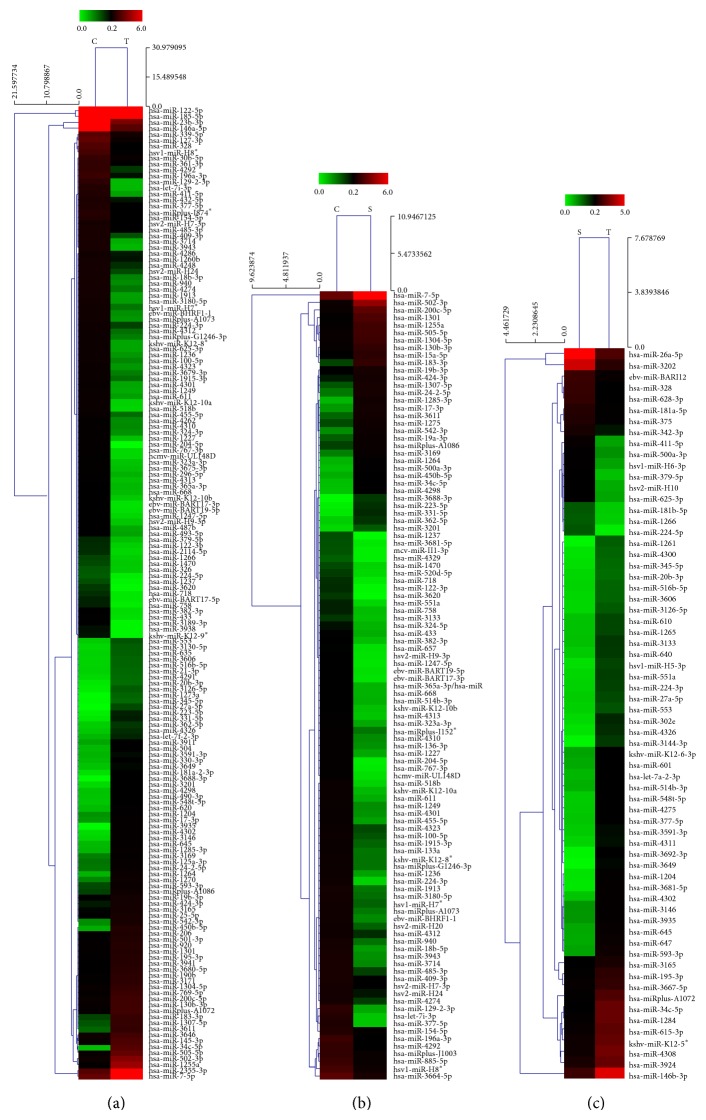
Heat map of the hierarchical clustering of 448 differentially expressed miRNAs in nonsmoker controls (C), smokers (S), and lung-cancer patients (T). (a) T versus C; (b) S versus C; (c) S versus T. Clustering is significant, with a *P* value of 0.001, according to Fisher's test. The miRNAs are shown on the *y*-axis and the groups are shown on the *x*-axis. Red indicates high expression and green indicates low expression of miRNAs.

**Figure 2:**
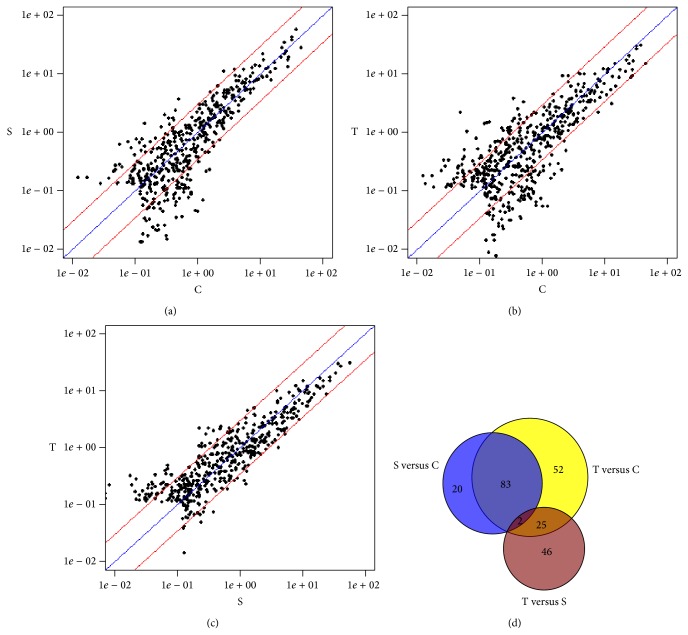
Differential expression profiles of 448 miRNAs in nonsmoker controls (C), smokers (S), and lung-cancer patients (T). (a) Scatter plot of S versus C; (b) scatter plot of T versus C; (c) scatter plot of T versus S. Central diagonal line and two outer diagonal lines in each panel represent the unchanged line and the threefold variation intervals line, respectively; (d) Venn diagram of the 448 differentially expressed miRNAs. Blue represents the differentially expressed miRNAs in S versus C; yellow represents the differentially expressed miRNAs in T versus C; brown represents the differentially expressed miRNAs in T versus S. 85 miRNAs were dysregulated in both S versus C and T versus C. The smallest differences in miRNA expression were in the comparisons T versus S and S versus C.

We determined the serum miRNA expression in 10 nonsmokers, 10 smokers, and 10 lung-cancer patients using the miRCURY LNA microRNA array. According to the manufacturer's recommendations, we set up the screening criteria for the differential expression of miRNAs as follows: (a) the average normalized intensities were less than 50; (b) the change in expression was more than threefold. In this way, 448 differentially expressed miRNAs were selected for the subsequent analyses. A heat map of the hierarchical clustering of the differentially expressed miRNAs is shown in Figure 1. Compared with the nonsmoker serum samples, the expression of 105 miRNAs was significantly altered in the serum samples of smokers. The expression of 32 and 73 candidate miRNAs was upregulated and downregulated, respectively (Figure 2(a)). Compared with the nonsmoker serum samples, the expression levels of 163 miRNAs were significantly altered in the lung-cancer patients. The expression of 74 and 89 candidate miRNAs was upregulated and downregulated, respectively (Figure 2(b)). Compared with the smokers, the expression of 73 candidate miRNAs was significantly altered in lung-cancer patients; 56 were upregulated and 17 downregulated (Figure 2(c)). As shown in Figure 2(d), compared with the nonsmokers' serum samples, 85 miRNAs were differentially expressed in both the lung-cancer patients' and smokers' serum samples. The 85 differentially expressed miRNAs accounted for 80.95% (85/105) of all differentially expressed miRNAs in the smokers and 52.15% (85/163) in the lung-cancer patients. Of these 85 miRNAs, the expression of 28 was upregulated and that of 57 was downregulated. To investigate the relationship between the serum miRNAs, smoking, and lung cancer, we screened these 85 miRNAs for candidate miRNAs.

### 3.2. Selection and Validation of Candidate Serum miRNAs

**Figure 3:**
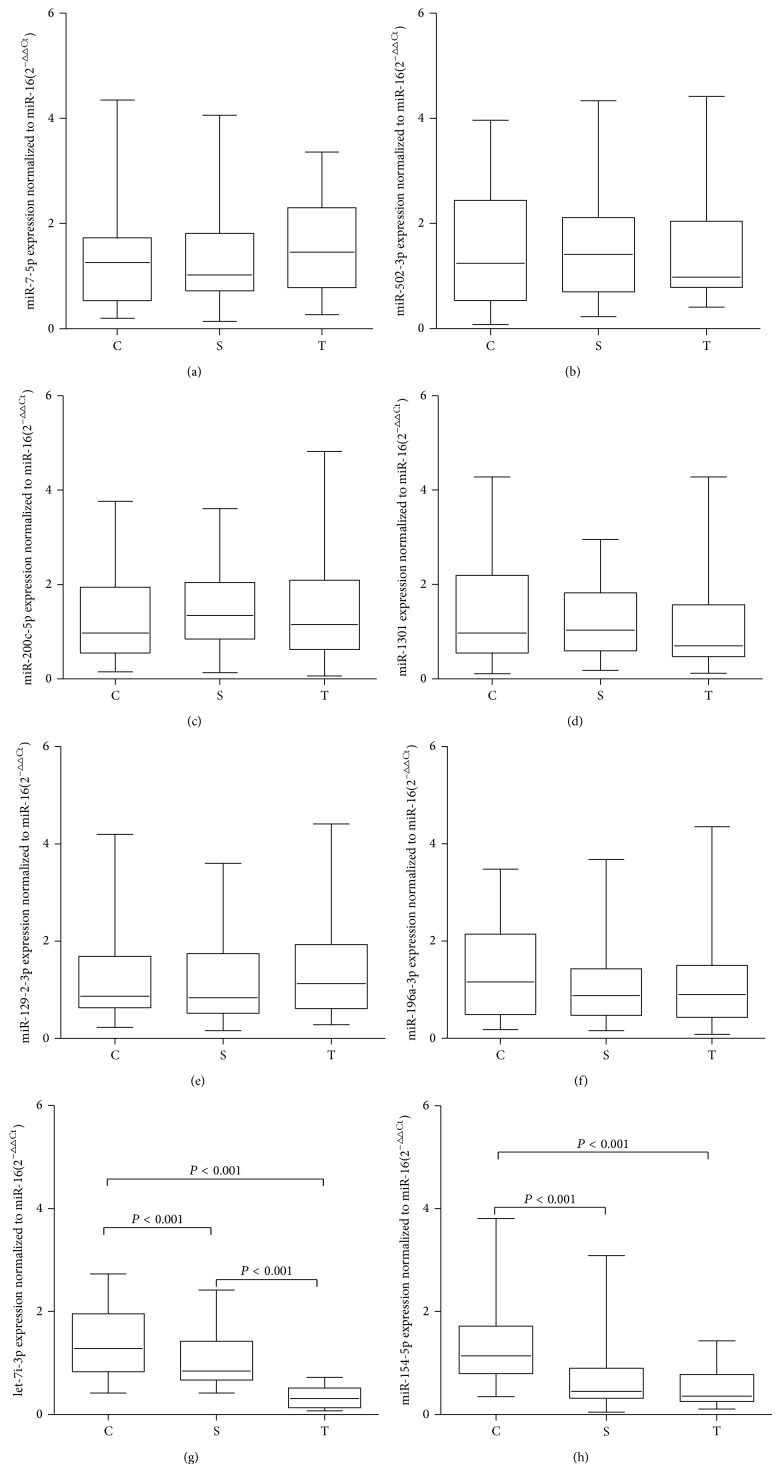
Expression levels of candidate miRNAs in the sera of nonsmoker controls (C), smokers (S), and lung-cancer patients (T). Box plots of the levels of miR-7-5p (a), miR-502-3p (b), miR-200c-3p (c), miR-1301 (d), miR-129-2-3p (e), miR-196a-3p (f), let-7i-3p (g), and miR-154-5p (h) in the sera of the nonsmoker controls (*n* = 30), smokers (*n* = 30), and lung-cancer patients (*n* = 30). The expression levels of the miRNAs are relative to the average levels of the controls and are normalized to miR-16. Statistically significant differences were analyzed with the Kruskal-Wallis test. The upper and lower limits of the boxes and the lines across the boxes indicate the 75th and 25th percentiles and the median, respectively.

Because miRNAs with low signal intensity are difficult to detect with qRT-PCR, we removed any miRNAs with low signal intensity from the above-mentioned 85 differentially expressed miRNAs. Eight miRNAs with high signal intensity were selected as candidate miRNAs for further study (shown in Table 2). Of these eight miRNAs, four (miR-7-5p, miR-502-3p, miR-200c-5p, and miR-1301) were upregulated and four (miR-129-2-3p, let-7i-3p, miR-196a-3p, and miR-154-5p) were downregulated in smokers and lung-cancer patients compared with the nonsmoking control serum samples, according to the results of the microarray analysis. To investigate whether the differential expression of these miRNAs can be attributed to cigarette smoke and lung cancer, we confirmed their expression with qRT-PCR in an independent sample set, consisting of 30 individual serum samples from each group (nonsmoking controls, smokers, and the lung-cancer patients). As shown in Figure 3, the levels of serums let-7i-3p (Kruskal-Wallis test, *P* < 0.05) and miR-154-5p (Kruskal-Wallis test, *P* < 0.05) were significantly downregulated in smokers and lung-cancer patients compared with those in the nonsmoking control serum samples. No significant differences were observed in the other six miRNAs (Kruskal-Wallis test, *P* > 0.05).

### 3.3. Serums let-7i-3p and miR-154-5p Are Associated with Smoking and Lung Cancer

**Figure 4:**
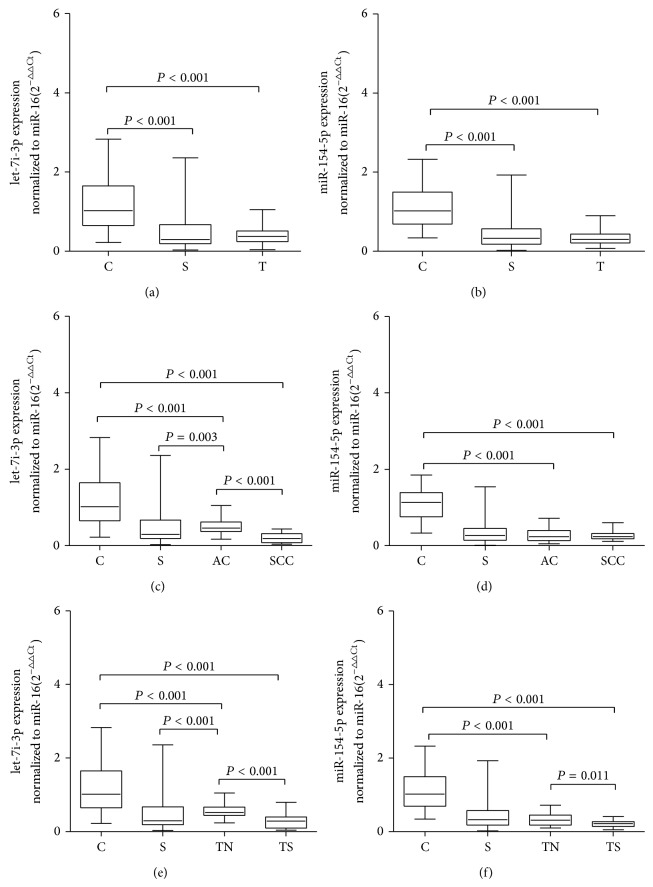
Serum levels of miRNAs let-7i-3p and miR-154-5p in nonsmoker controls (C), smokers (S), and lung-cancer patients (T). Box plots of the levels of let-7i-3p (a) and miR-154-5p (b) in the sera of nonsmoker controls (*n* = 65), smokers (*n* = 100), and lung-cancer patients (*n* = 84). The expression levels of let-7i-3p (c) and miR-154-5p (d) in lung-cancer patients with squamous cell carcinoma and adenocarcinoma (AC). The expression levels of let-7i-3p (e) and miR-154-5p (f) in the smoking lung-cancer group (TS) and the nonsmoking lung-cancer group (TN). The expression levels of the miRNAs are relative to the average values of the controls and are normalized to miR-16. Statistically significant differences were analyzed with the Kruskal-Wallis test. The upper and lower limits of the boxes and the lines across the boxes indicate the 75th and 25th percentiles and the median, respectively.

To confirm that serums let-7i-3p and miR-154-5p are associated with smoking and lung cancer, we quantified the levels of let-7i-3p and miR-154-5p in larger samples with qRT-PCR. We collected individual serum samples from 100 smokers, 84 lung-cancer patients, and 65 nonsmoking controls. As shown in Figures 4(a) and 4(b), compared with the nonsmoking control serum samples, the serum levels of both let-7i-3p and miR-154-5p were significantly downregulated in the smokers (Kruskal-Wallis test, *P* < 0.001) and lung-cancer patients (Kruskal-Wallis test, *P* < 0.001). However, the expression of serums let-7i-3p (Kruskal-Wallis test, *P* = 0.962) and miR-154-5p (Kruskal-Wallis test, *P* = 0.290) did not differ significantly between the lung-cancer patients and the smokers.

To explore the relationships between these two miRNAs (let-7i-3p and miR-154-5p), the pathological type of lung cancer, and smoking, we compared the levels of let-7i-3p and miR-154-5p in 84 serum samples from lung-cancer patients, according to their pathological types and smoking characteristics. The lung-cancer subtypes were classified according to the World Health Organization classification and the International Union against Cancer staging system. The 84 samples of the lung-cancer patients were grouped into 33 cases of squamous cell carcinoma and 51 cases of adenocarcinoma. The 84 lung-cancer patients included 53 smokers and 31 nonsmokers. The detailed characteristics of the lung-cancer patients are shown in Table 1. Their ages ranged from 45 to 72 years. The mean ages of the three groups did not differ significantly.

As shown in Figures 4(c) and 4(d), the expression levels of serums let-7i-3p (Kruskal-Wallis test, *P* < 0.001) and miR-154-5p (Kruskal-Wallis test, *P* < 0.001) were significantly downregulated in the samples of patients with squamous cell carcinoma and adenocarcinoma compared with the nonsmoking controls. The serum levels of let-7i-3p were downregulated about threefold in the patients with adenocarcinoma and about sevenfold in the patients with squamous cell carcinoma. The serum levels of miR-154-5p were downregulated about fourfold in both the adenocarcinoma and the squamous cell carcinoma patients. There was no statistically significant difference in miR-154-5p expression between the squamous cell carcinoma group and the adenocarcinoma group among the current smokers. Compared with the smokers, there was no significant difference in let-7i-3p expression in squamous cell carcinoma samples, but the level of let-7i-3p in the adenocarcinomas was significantly upregulated (Kruskal-Wallis test, *P* = 0.003).

When the lung-cancer patients were classified into the smoking group (TS) and the nonsmoking group (TN) and compared with the nonsmoking control group, let-7i-3p (Kruskal-Wallis test, *P* < 0.001) and miR-154-5p (Kruskal-Wallis test, *P* < 0.001) were both significantly downregulated in the TS group and TN group. However, as shown in Figures 4(e) and 4(f), let-7i-3p (Kruskal-Wallis test, *P* < 0.001) and miR-154-5p (Kruskal-Wallis test, *P* = 0.011) were significantly downregulated in the TS group compared with the TN group. These results indicate that the downregulation of serums let-7i-3p and miR-154-5p was associated with smoking and lung cancer.

**Figure 5:**
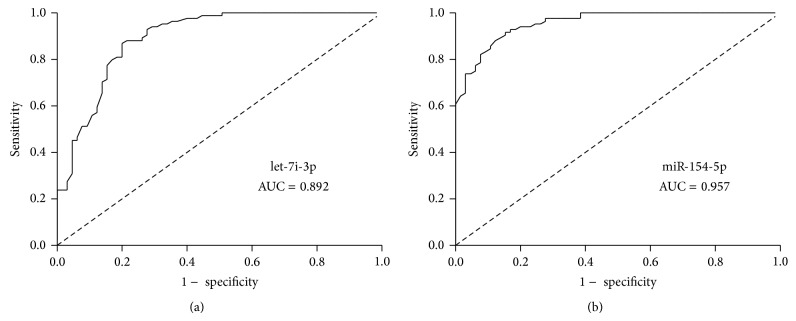
Evaluation of serums let-7i-3p and miR-154-5p as potential biomarkers of smoking-related lung cancer. Receiver operating characteristic (ROC) curve analyses of serums let-7i-3p (a) and miR-154-5p (b) were performed to discriminate patients with smoking-related lung cancer from nonsmokers. The AUCs for serums let-7i-3p and miR-154-5p were 0.892 and 0.957, respectively.

A ROC curve analysis was performed to evaluate the sensitivity and specificity of let-7i-3p and miR-154-5p as biomarkers of smoking-related lung cancer. The AUCs for serums let-7i-3p and miR-154-5p were 0.892 and 0.957, respectively (Figures 5(a) and 5(b)). These results indicate that serums let-7i-3p and miR-154-5p levels can potentially be used as biomarkers to distinguish smoking-related lung-cancer patients from nonsmoking controls.

### 3.4. Dose-Response Relationship between Exposure to Cigarette Smoke and Serum Levels of let-7i-3p and miR-154-5p

**Figure 6:**
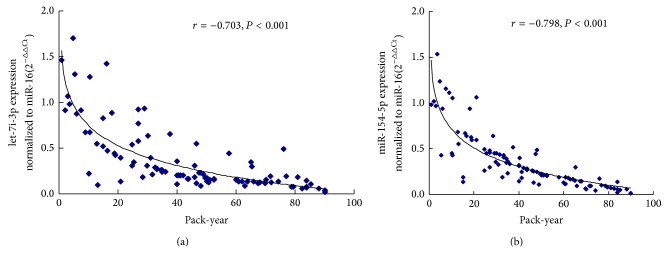
Scatter plots of the correlation between serums let-7i-3p (a) and miR-154-5p (b) levels and the smoking index. The relative expression levels of the serum miRNAs were normalized to miR-16. The correlations between the serums let-7i-3p (*r* = −0.703, *P* < 0.01) and miR-154-5p (*r* = −0.798, *P* < 0.01) levels and the smoking index were analyzed using Pearson's bivariate correlation.

To confirm that exposure to cigarette smoke induces changes in the expression of serum miRNAs, we measured the smoking indices of 100 smokers and analyzed the relationship between cigarette smoking and the serum levels of let-7i-3p and miR-154-5p. The expression of serums let-7i-3p and miR-154-5p was significantly lower in smokers. As shown in Figure 6, Pearson's bivariate correlation analysis of the smoking index against the relative expression of let-7i-3p (*r* = −0.703, *P* < 0.01) and miR-154-5p (*r* = −0.798, *P* < 0.01) showed significantly negative correlations, such that an increase in the smoking index was associated with lower relative expression levels of let-7i-3p and miR-154-5p.

### 3.5. Predicted Functions of let-7i-3p and miR-154-5p in Lung Carcinogenesis

According to our findings, let-7i-3p and miR-154-5p are both significantly downregulated in the sera of current smokers and lung-cancer patients. These results suggest that let-7i-3p and miR-154-5p play important roles during cigarette-smoking-induced lung carcinogenesis. To explore the potential functions of let-7i-3p and miR-154-5p, we used three computational programs to predict their target genes.

After systematically analyzing the predicted target genes, we identified the 52 most promising potential target genes for let-7i-3p and the seven most promising potential target genes for miR-154-5p. As shown in Table 3, the main target genes of let-7i-3p and miR-154-5p included* ACTB*,* ATP2A2*,* BDNF*,* BTG3*,* ROS*,* ATG7*, and* CUL2*. A pathway analysis showed that these target genes are involved in the NF-*κ*B, HIF-1, MAPK, Notch, and autophagic molecular signaling pathways. These target genes and signaling pathways play important roles in the occurrence, development, and metastasis of lung cancer. Collectively, these predictions and analyses imply that let-7i-3p and miR-154-5p potentially function during cigarette-smoking-induced lung carcinogenesis.

## 4. Discussion

According to previous studies, circulating miRNAs are potential biomarkers for a variety of cancers, including NSCLC [12–15, 27], and cigarette smoking can alter the plasma miRNA profiles of healthy subjects [22]. However, the relationship between circulating miRNAs and cigarette-smoke-induced lung cancer is still unclear. In view of the known etiological relationship between smoking and lung cancer, we explored the changes in serum miRNAs in smokers and lung-cancer patients in the present study.

A microRNA array showed that cigarette smoke causes changes in serum miRNA expression. A clustering analysis of the differentially expressed miRNAs demonstrated that serum miRNA profiles can distinguish smokers and lung-cancer patients from nonsmokers. The levels of 105 miRNAs were differentially expressed threefold in the sera of smokers compared with the sera of nonsmoking controls, and the expression of 69.52% (73/105) of these miRNAs was downregulated. Several previous studies have demonstrated that exposure to cigarette smoke results in a marked downregulation of miRNA expression in the lungs of both mice and rats [19, 21]. According to Schembri et al., smoking causes the expression of many miRNAs to be downregulated in the bronchial epithelium [20]. Most of these miRNAs are reduced in smokers, indicating that the overall suppressive effect of miRNAs on gene transcription and translation is reduced by smoking. The downregulation of several miRNAs was also observed in the lungs of rats treated with typical components of cigarette smoke, such as the tobacco-specific nitrosamine [28]. These previous studies provide evidence that miRNA dysregulation is an important mechanism in cigarette-smoke-induced lung carcinogenesis. The present study shows that the dysregulation of miRNAs occurs in the sera of smokers, providing further evidence at the serum level.

Further validation and screening demonstrated that the expression of let-7i-3p and miR-154-5p is significantly downregulated in the sera of smokers and lung-cancer patients. A correlation analysis showed a negative correlation between the smoking index and the serum levels of let-7i-3p and miR-154-5p and that an increasing trend in the smoking index is associated with lower relative expression of let-7i-3p and miR-154-5p. A previous study also demonstrated that the expression of many miRNAs was downregulated in lungs of rats exposed to cigarette smoke [19]. An analysis of differential miRNA expression revealed that let-7c was significantly downregulated in the sputum of currently smoking patients with chronic obstructive pulmonary disease [29]. These findings provide evidence that exposure to cigarette smoke correlates negatively with miRNA expression. Our results also indicate that exposure to cigarette smoke induces changes in serum miRNAs and is associated with the levels of serum miRNAs.

We performed a correlation analysis to investigate the similarity in the serum miRNA profiles of smokers and lung-cancer patients. Of the differentially expressed miRNAs, 85 were differentially expressed in the serum samples of both lung-cancer patients and smokers. Continuous changes in these serum miRNAs were observed from nonsmoking controls to smoking males and lung-cancer patients. Serum miRNAs are increasingly recognized as stable biomarkers of lung cancer, with utility in its diagnosis. Keller and Nolen reported that circulating miRNAs are stable in serum and could be used as potential biomarkers for the diagnosis of noninvasive lung cancer [30, 31]. In the present study, a ROC curve analysis showed that the AUCs for serums let-7i-3p and miR-154-5p were 0.892 and 0.957, respectively. These results indicate that let-7i-3p and miR-154-5p are potential biomarkers and play important roles during exposure to cigarette smoke and cigarette-smoking-induced lung carcinogenesis. The downregulated expression of serums let-7i-3p and miR-154-5p was also related to the histological subtype of the lung cancer and the smoking history of the patient. According to previous studies, although there is accumulating evidence of miRNAs in the blood and serum, the origin of these circulating extracellular miRNAs remains unclear. It has been reported that tumor-derived miRNAs are probably packaged in exosomes or microvesicles and released into the blood to regulate their target genes [16, 32]. Therefore, it is necessary to clarify the origins and functions of serums let-7i-3p and miR-154-5p in future studies.

In this study, the levels of let-7i-3p and miR-154-5p were significantly downregulated in the sera of smokers and lung-cancer patients. Interestingly, the levels of these two miRNAs were significantly lower in the sera of the smoking lung-cancer group than in the nonsmoking lung-cancer group. let-7i-3p is a member of the let-7 family, which regulates many important lung-cancer-associated oncogenes and inhibits the growth of lung-cancer cell lines* in vitro* and* in vivo* [33, 34]. The miR-154 family has been implicated in lung development and several diseases, including pulmonary fibrosis and cancer [35, 36]. Therefore, we predicted the potential target genes of let-7i-3p and miR-154-5p and their involvement in various cellular pathways. Many studies have shown that these target genes play important roles in the occurrence, development, and metastasis of lung cancer [37–43]. A pathway analysis showed that they may play these roles through cellular pathways such as the NF-*κ*B, focal adhesion, MAPK, Notch, HIF-1, and calcium signaling pathway. We speculate that let-7i-3p and miR-154-5p are associated with the development or progression of cigarette-smoke-induced lung cancer. However, further studies are required to clarify the functions of let-7i-3p and miR-154-5p in cigarette-smoke-induced lung carcinogenesis.

## 5. Conclusions

In summary, we profiled the global serum miRNA expression in nonsmoking controls, smokers, and lung-cancer patients. Our findings suggest that cigarette smoking causes most serum miRNAs to be downregulated. The serum miRNA expression profiles of lung-cancer patients showed a trend similar to that in smokers. Serums let-7i-3p and miR-154-5p, which correlated negatively with the smoking index, may be potential biomarkers of exposure to cigarette smoke and cigarette-smoke-induced lung cancer. The downregulated expression of serums let-7i-3p and miR-154-5p is probably related to the pathological type of the cancer and the smoking history of the patient. Target gene prediction and pathway analysis of let-7i-3p and miR-154-5p suggested that these miRNAs are involved in regulating the occurrence, development, and metastasis of NSCLC through multiple target genes and signaling pathways. These findings suggest that let-7i-3p and miR-154-5p are associated with the development or progression of cigarette-smoke-induced lung cancer.

## Figures and Tables

**Table 1:** Baseline characteristics of the nonsmoker controls (C), smokers (S), smoking lung-cancer group (TS), and nonsmoking lung-cancer group (TN).

Group	Smokers	Nonsmokers	Age (years)∗	Age when started smoking (years)	Number of cigarettes per day	Number of years smoked (years)	Pack-years	Adenocarcinoma	Squamous cell carcinoma
C	—	65	55.0 ± 10.2	—	—	—	—	—	—
S	100	—	53.9 ± 10.3	29.6 ± 8.2	34.1 ± 14.6	24.2 ± 9.5	43.3 ± 24.4	—	—
TS	53	—	56.5 ± 9.4	27.2 ± 8.2	39.0 ± 9.2	29.3 ± 9.8	56.4 ± 14.1	23	30
TN	—	31	56.7 ± 12.9	—	—	—	—	3	28

^*^The means and standard deviations are shown for age (years) and smoking status (pack-years).

**Table 2:** Candidate miRNAs selected from the miRNAs with differential expression profiles on a microarray analysis.

Name of miRNA	(S versus C∗) Fold change	Foreground-background^§^		(T versus C) Fold-change	Foreground-background	
		C	S		C	T
Upregulated						
hsa-miR-7-5p	4.26	1045	4638	3.53	1045	4214
hsa-miR-502-3p	7.05	206	1511	6.33	206	1488
hsa-miR-200c-5p	5.34	178	989	3.72	178	754
hsa-miR-1301	6.52	122	832	3.42	122	478
Downregulated						
hsa-miR-154-5p	3.4	419	128	3.96	419	120
hsa-let-7i-3p	23.05	410	18.5	18.71	410	25
hsa-miR-129-2-3p	4.95	28	97	22.59	459	25
hsa-miR-196a-3p	6.81	626	95	8.25	626	86

^*^C, S, and T represent nonsmoker controls, smokers, and lung-cancer patients, respectively.

^§^Foreground-background represents the signal of the probe after background correction.

**Table 3:** Prediction of the target genes of let-7i-3p and miR-154-5p and their pathways.

miRNA	Target gene	Signaling pathway
let-7i-3p^§^	*ACTB *	Phagosome, Hippo signaling pathways
	*ADHFE1 *	Hippo signaling pathway
	*ATP2A2 *	Calcium signaling pathway
	*BDNF *	MAPK signaling pathway
	*BTG3 *	RNA degradation
	*EMP2 *	VEGF signaling pathway
	*DLX5 *	IRS-2-AKT signaling pathway
	*CXCR6 *	Chemokine signaling pathway
	*CXCL12 *	Chemokine signaling pathway, NF-*κ*B signaling pathway
	*HES1 *	Notch signaling pathway
	*RASSF3 *	Pathways in cancer, NSCLC
	*SIX1 *	Transcriptional misregulation in cancer

miR-154-5p∗	*ABCC9 *	ABC transporters
	*ROS *	Transcriptional misregulation in cancers, ErbB signaling pathway, focal adhesion, NF-*κ*B signaling pathways
	*ATG7 *	Cellular processes, regulation of autophagy
	*TNFAIP3 *	NF-*κ*B signaling pathway, NOD-like receptor signaling pathway
	*CUL2 *	HIF-1 signaling pathway, pathways in cancer

^§^Target gene prediction implied that the main target genes of let-7i-3p are *ADHFE*1, *ACTB*, *ATP2A2*, and so forth, which play roles in the occurrence, development, and metastasis of lung cells during carcinogenesis through the NF-*κ*B, MAPK, and Notch signaling pathways.

∗Target gene prediction implied that the main target genes of miR-154-5p are *ABCC9*, *ROS*, *ATG7*, and so forth, which play roles in the occurrence, development, and metastasis of lung cells during carcinogenesis through the NF-*κ*B, HIF-1, MAPK, and autophagy signaling pathways.
